# Reducing Conditions Favor Magnetosome Production in *Magnetospirillum magneticum* AMB-1

**DOI:** 10.3389/fmicb.2019.00582

**Published:** 2019-03-29

**Authors:** Agata Olszewska-Widdrat, Gabriele Schiro, Victoria E. Reichel, Damien Faivre

**Affiliations:** ^1^Max Planck Institute of Colloids and Interfaces, Science Park Golm, Potsdam, Germany; ^2^Aix-Marseille University, CEA, CNRS, BIAM, Saint-Paul-lés-Durance, France

**Keywords:** redox potential, magnetotactic bacteria, iron, magnetite, biomineralization

## Abstract

Magnetotactic bacteria (MTB) are a heterogeneous group of Gram-negative prokaryotes, which all produce special magnetic organelles called magnetosomes. The magnetosome consists of a magnetic nanoparticle, either magnetite (Fe_3_O_4_) or greigite (Fe_3_S_4_), embedded in a membrane, which renders the systems colloidaly stable, a desirable property for biotechnological applications. Although these bacteria are able to regulate the formation of magnetosomes through a biologically-controlled mechanism, the environment in general and the physico–chemical conditions surrounding the cells in particular also influence biomineralization. This work thus aims at understanding how such external conditions, in particular the extracellular oxidation reduction potential, influence magnetite formation in the strain *Magnetospirillum magneticum* AMB-1. Controlled cultivation of the microorganisms was performed at different redox potential in a bioreactor and the formation of magnetosomes was assessed by microscopic and spectroscopic techniques. Our results show that the formation of magnetosomes is inhibited at the highest potential tested (0 mV), whereas biomineralization is facilitated under reduced conditions (-500 mV). This result improves the understanding of the biomineralization process in MTB and provides useful information in sight of a large scale production of magnetosomes for different applications.

## Introduction

Magnetotactic bacteria (MTB) are a diverse group of prokaryotes able to synthesize magnetic nanoparticles in organelles called magnetosomes. Magnetosomes are composed of a magnetic core, nanocrystals of either magnetite or greigite, encapsulated in a lipid bilayer (Uebe and Schuler, 2016). The nanoparticles are organized in chains due to a protein-based backbone stabilizing the alignment and enabling the bacteria to align along the Earth’s magnetic field lines (Faivre and Schüler, 2008; Faivre, 2015). Living cells have found applications as drug carriers or as biohybrids thanks to their magnetic behavior (Taherkhani et al., 2014; Felfoul et al., 2016; Stanton et al., 2017). In addition, the unique characteristics of magnetosomes enable applications such as contrast agent in magnetic resonance imaging. Magnetosomes can also be functionalized with proteins or antibodies and used as biocatalysts in various chemical applications (Yoshino et al., 2009; Ginet et al., 2011; Taukulis et al., 2015).

The chemical process controlling magnetite formation in magnetosomes is under study, specifically because size and morphology control as well as colloidal stability is difficult to obtain in purely chemical syntheses. The chemical stability of magnetite is restricted to a limited domain in the oxidation-reduction potential (ORP)/pH space where magnetite formation typically takes place at ORP values from -200 to -400 mV and pH > 8 (Faivre and Schüler, 2008). Nevertheless, MTB are able to provide chemical conditions suitable for the production of the appropriate iron phase under different physiological conditions and therefore, to biomineralize magnetite under adverse circumstances. While there are still a lot of uncertainties concerning their physical and chemical conditions leading to biomineralization, the genetic background has been the subject of numerous studies highlighting the genetic control on the process (Uebe and Schuler, 2016). A genomic island, called Magnetosome Island (MAI) was identified containing the genes (*mam* and *mms* genes) responsible for crystal formation (Ullrich et al., 2005). The process can also be summarized as follows: (1) Iron is taken up both as Fe (II) and Fe (III) (Faivre et al., 2007); (2) The cytoplasmic membrane forms an invagination, which will then acts as the chemical reactor or the formation of magnetosomes (Komeili et al., 2006). (3) Iron is transported inside the membrane by the iron transport proteins such as MamM, MamB, FeoA and FeoB (Uebe and Schuler, 2016). (4) Iron is then partially reduced and precipitated as magnetite (Baumgartner et al., 2013; Siponen et al., 2013; Amor et al., 2018). In this process, some magnetosome-associated proteins, such as MamE, MamP, MamT, and MamX, take an active part in the control of the Fe^2+^ to Fe^3+^ ratio (Siponen et al., 2013; Jones et al., 2015; Barber-Zucker et al., 2016). In addition to iron, oxygen is also a particularly important redox-active player affecting bacterial growth and magetosome formation (Heyen and Schüler, 2003; Liu et al., 2010; Yang et al., 2013). Firstly, O_2_ coming from the air was speculated to take an active part in the process, due to the competition between biomineralization process and respiration (Blakemore et al., 1985) before isotope analyses demonstrated that the oxygen involved in the magnetite biomineralization comes from water (Mandernack et al., 1999). Here, we use *Magnetospirillum magneticum* ABM-1, a facultative anaerobe capable of growing in the presence or in the absence of oxygen. However, different respiration pathways are being used while the final electron acceptor is the O_2_ during aerobic growth.

The general relationship between MTB and their extracellular environment have been explored aside from redox parameters. Typically, these experiments have investigated potential changes in the morphology of the cells grown in different physical and chemical conditions, as well as changes in the biomineralization process caused by extracellular disturbances. While differences in physical parameters such as temperature or magnetic field have shown only marginal and weak effects, changes in the extracellular chemical environment seem to have a larger influence on the formation of magnetosomes (Faivre et al., 2008; Moisescu et al., 2014). For example, change in the pH of the growth medium impacts the iron uptake, and resulted in altered morphologies of the produced crystals (Moisescu et al., 2014). The initial Fe availability causes change in crystal size distribution, morphology and aspect ratio (Faivre et al., 2008). Oxygen inhibits the formation of magnetosomes at high O_2_ partial pressures (Heyen and Schüler, 2003). Accordingly, smaller magnetosomes are formed such that the magnetic properties of magnetosomes and MTB indirectly dependent on dissolved oxygen concentration (Li and Pan, 2012). Oxygen concentration is ecologically relevant for MTB, since their natural habitat is found at the oxic-anoxic transition zone (OATZ), where chemical gradients are present (Lefèvre and Bazylinski, 2013). In natural waters, such as lakes and ponds, a chemical gradient is created in the sediments layer by the diffusion of oxygen from the water surface downward, and hydrogen sulfide produced by the sulfate reducing bacteria, which diffuses upward from the anaerobic zone. MTB are thought to have developed their complex magnetic apparatus in order to find the exact position in these chemical gradients (Lefèvre et al., 2014). Both the observed effect of oxygen on biomineralization and the ecological behavior of MTB suggest a strong connection between ORP and magnetosome formation. Oxidation – reduction potential is an indicator of electrochemical activity of substances in environmental conditions. ORP is defined as a measure of the intensity of its oxidizing and reducing properties.

So far, the effect of ORP was only indirectly assessed by changing the partial pressure of oxygen during cultivation (Heyen and Schüler, 2003). Decorrelating redox conditions from oxygen concentration is yet to be done. Here, we thus conducted a study where the cells were grown in a bioreactor, where pH, temperature, ORP and pO_2_ were continuously monitored and kept stable trough feedback systems. We used titanium (III) citrate as reducing agent and potassium ferricyanide as oxidizer. Titanium (III) citrate was already utilized as a reductive compound for microorganism growth (Jones and Pickard, 1980). Potassium ferricyanide, in turn, was also used in previous redox control of *Escherichia coli* anaerobic fermentative growth (Bagramyan et al., 2000). In order to check the influence of ORP on magnetosome formation, we investigated the anaerobic growth of *Magnetospirillum magneticum* strain AMB-1 (Matsunaga et al., 1991) at neutral pH (7), and varied the ORP from reduced (ORP = – 500 mV) to neutral (ORP = 0 mV) compared to a control experiment where ORP was not regulated but continuously measured.

## Materials and Methods

### Bacterial Strains, Flask Growth, Cell Density Measurements, and Toxicity Tests

*M. magneticum* AMB-1 strain was purchased from the American Collection of Cell Cultures (No. 70264). The bacteria were grown in a modified flask standard medium (FSM) containing (per liter deionized water) 0.1 g KH_2_PO_4_ (Acros organics, Belgium), 0.15 g MgSO_4_ × 7 H_2_O, 2.38 g Hepes, 0.34 g NaNO_3_ (Sigma-Aldrich, United States), 0.1 g yeast extract, 3 g soya peptone, and 100 μM iron (III) citrate (Alfa Aesar, Germany). If not otherwise specified, all chemicals were purchased from Carl Roth (Karlsruhe, Germany). The medium contained 27 mM sodium pyruvate (Alfa Aesar, Germany), modified trace elements, and an iron–depleted mineral solution (Widdel and Back, 1992). Afterward, the flasks were sealed with open ended caps and rubber stoppers and autoclaved. Inoculation was done with 10% (v/v) subculture and incubated under gentle shaking (100 rpm) at 28°C for 24–48 h. All of the experiments were performed with axenic conditions, with regular microscopic controls. A UV-spectrophotometer (Nanophotometer, IMPLEN, Germany) was used to determine the cell growth by measuring the optical density at 565 nm (OD_565_). The optical density was measured with two bar magnets (Supermagnete, Germany) placed parallel (OD_min_) and perpendicular to the light beam (OD_max_). The coefficient of magnetically induced differential light scattering (C_mag_), describing the magnetic orientation of the cells was calculated following a published procedure (Schüler et al., 1995).

In order to correlate OD_565_ values with number of cells, cell-counting experiments were performed using a Neubauer Chamber (Brand, Germany) with an optical microscope (AXIO-microscope, Zeiss, Germany) and anaerobic cultures. The cell density was measured to be 9.07 × 10^7^ cells mL^-1^, at an OD_565_ of 0.277. A linear correlation of cell count with the optical density was assumed, as done in other studies on *Magnetospirillum* (Heyen and Schüler, 2003) such that cell density of 3.27 × 10^8^ cells × mL^-1^ corresponded to an OD_565_ of 1. This cell density value was used for further normalization of iron content in cells and it is similar to cell density reported earlier (Heyen and Schüler, 2003). The results were compared to the cell dry mass using the value provided in the literature (Heyen and Schüler, 2003), which reported a culture density at OD_565_ = 1, a dry weight of 0.28 g.

We used titanium (III) citrate as reducing agent and potassium ferricyanide as oxidizer. For Titanium (III) citrate, no major toxicity was reported for an anaerobic bacterium such as *Clostridium formicoaceticum* or for a facultative anaerobic bacterium such as *Pseudomonas denitrificans* (Zehnder and Wuhrmann, 1976). As toxicity tests, the concentration of titanium (III) citrate was tested on *M. magneticum* AMB-1 cells in flasks with concentrations varying from 0.22 to 15 mM. Potential toxic effects of potassium ferricyanide were also measured at different concentrations (0.1 mM < [C_6_N_6_FeK_3_] < 3.6 mM). The experiments were conducted in 250 ml flasks filled FSM and treated as described above. To quantify the cell growth, OD_565_ was measured directly after the inoculation, and after 24 h.

### Growth in the Bioreactor and ORP Determination

Before testing the ORP conditions, non-magnetic precultures were prepared as previously described (Faivre et al., 2007), with the following modifications: from a fully grown microaerobic FSM iron-rich culture, 3% (v/v) was inoculated into 100 mL of fresh iron–depleted culture medium. The entire culture was then used as subsequent preculture for flasks containing 1 L of iron-depleted fresh medium. The final, 1 L subculture was used for the experiments performed in the bioreactor. The age of the preculture was tested in order to better understand the following cellular growth in the bioreactor. Experiments did not show differences in growth between 1, 2, and 3 days-old inoculum (see Supplementary Figure S1). Forty eight hours old precultures were consequently chosen as inoculum for further experiments in the bioreactor. All of the precultures and taken samples were controlled for any potential contaminants by regular checks, using the optical microscope. Moreover the C_mag_ was measured to distinguish between magnetic and non-magnetic cells.

The bacteria were grown in the bioreactor with a volume of 16 L (L1523, Bioengineering, Switzerland). The bioreactor was able to keep constant the following parameters: temperature, oxygen concentration, pH and the steering speed (Supplementary Figure S3). The bioreactor was filled with 12 L of FSM medium, omitting Hepes; sodium pyruvate was substituted by aqueous solution of 0.3% (v/v) of L-(+)-lactic acid (Sigma-Aldrich, United States). The pH of the culture was controlled by the addition of either 1 M NaOH or 0.5 M H_2_SO_4_. The ORP control was obtained by the addition of either 0.8 M of titanium (III) citrate or 0.3% (w/v) potassium ferricyanide solution. The oxygen concentration was continuously measured using an oxygen probe (Bioengineering, Switzerland). After sterilization of the filled reservoir (121°C for 35 min), the syringe providing nitrogen source was plugged in during the cooling process to sparge nitrogen through the medium and obtain oxygen-free conditions. After the desired temperature was reached, the pH and the ORP values were adjusted. As such, the bioreactor was prepared to be inoculated and to perform the experiments. All experiments were done in triplicates and error bars are reported as standard error of the mean. Samples were taken after 4, 14, 16, 18 20, 22, and 24 h after the beginning of the growth in the bioreactor. Sampling was done in the proximity of a flame utilizing a sterile syringe. 20 mL of culture was removed each time, from which the OD_565_ was first measured. Sixteen microliter cultures were then centrifuged at 8000 rpm for 10 min at 4°C (Avanti J-E centrifuge, Beckman Coulter, United States), pellets and supernatant were divided and stored at -20°C until further analysis. The remaining 4 mL were used for analyses using electron and optical microscopies. The ORP value of the suspension was measured using platinum redox electrode (Pt4805-DPAS-SC**-**K8S METTLER TOLEDO).

### Iron Concentration Measurements

Iron measurements were made using an inductively-coupled plasma optical emission spectrometer (ICP OES, Optima 8000, PerkinElmer Inc., United States). For iron analysis, the samples containing bacterial pellets were dissolved in 500 μL of an *aqua regia* solution [65% w/v HNO_3_ and 35% w/v HCl, in a ration 1:2 (v/v)]. The samples were incubated overnight in glass vials at 40 °C in order to dissolve the iron species.

### Optical Microscopy

The cells length was measured at time 0 and at time 24 h for each experiment. Images were taken using an optical microscope (Imager A2 AXIO-microscope, Carl Zeiss, Germany) at 39.69 X magnification. The length of 100 cells per condition was then measured using the software “Image J” (National Institute of Health, United States). A straight line was drawn from the one end to the other end of each cell, and this line was then measured (Supplementary Figure S2A).

### TEM Analysis

For electron microscopy, *M. magneticum* cells were concentrated by centrifugation (5000 rpm, 5 min, 4°C), adsorbed onto a cooper grid, rinsed once with buffer (10 mM Hepes, 5 mM EDTA) and once with distilled water. The samples were imaged using a Zeiss 912 Omega transmission electron microscope at 120 kV.

The transmission electron microscopy images were analyzed with “ImageJ” in order to determine the size of magnetosomes, their amount per cell and the cell length (Supplementary Figures S2B–F). The magnetosomes dimensions were estimated by calculating the best fit of ellipse to the contours (Devouard et al., 1998).

### Magnetosome Size Analysis Using X-Ray Diffraction

To complement the magnetosomes size evaluation by TEM, X-ray diffraction (XRD) analyses were performed. XRD measurements were performed at the μ-Spot beam line of the BESSY II synchrotron facility of the Helmholtz Center for Energy in Berlin, Germany (Paris et al., 2007). The energy was set up to 15 keV (λ = 0.82656 Å) with a Si 111 monochromator. For measurements, a thin Kapton foil (Breitlander GmbH, Hamm, Germany, Cat. No. CH-440) was clamped on a special custom-made sample holder (Schmitz, 2010; Fischer et al., 2011). Ten microliter of each sample solution were pipetted on the thin Kapton film. The samples were mixed with the internal α-quartz standard (Standard Reference Material, 1878, NIST) prior to drying. After drying on the Kapton foil, the samples were measured with a 100 μm beam. The diffraction data were acquired on a MarMosaic 225 detector (Mar USA, Evanston, United States), consisting of nine independent CCD cameras. The detector resolution was 3072 × 3072 pixels, whereas the pixel size was 73.242 μm. For monitoring the XRD, the Spec program (SPEC, 2010) was used. Exposure times varied between 5 and 300 s, depending on the intensity of the signal. Each sample was measured three times. The sample to detector distance was set to 150–170 mm. The software DPDAK was used to fit the peaks with a pseudo-Voigt function. The internal α-quartz standard was used to calculate the exact sample-to-detector distance and the instrumental peak broadening. The crystallite size of the magnetosomes was then calculated from the corrected full width at half maximum (FWHM_corr_) of the (311)-peak of magnetite (Benecke et al., 2014) based on the Scherrer equation (Equation 1) (Widdrat et al., 2017).

(1) $$\begin{array}{c} crystallite\;site\;=\;\frac{2\pi }{FWHM_{corr}} \end{array}$$

## Results

### Toxicity Tests

**FIGURE 1:**
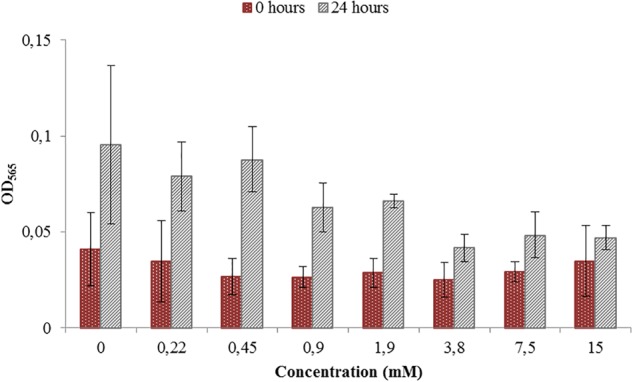
Toxicity test for a range of Titanium (III) citrate concentrations used during *M. magneticum* cultivation. The AMB-1 cultures were grown in 500 mL flasks bubbled with N_2_ in order to sustain anaerobic conditions. Ten milliliter of the preculture was used as an inoculum for each flask. Cultures without Titanium (III) citrate served a control. Optical density (OD_565_) was measured for each concentration immediately after the inoculation (t0, brown bars) and after 24 h of cell cultivation (t24, gray bars). Error bars represent the standard deviation of the three repetitions.

**FIGURE 2:**
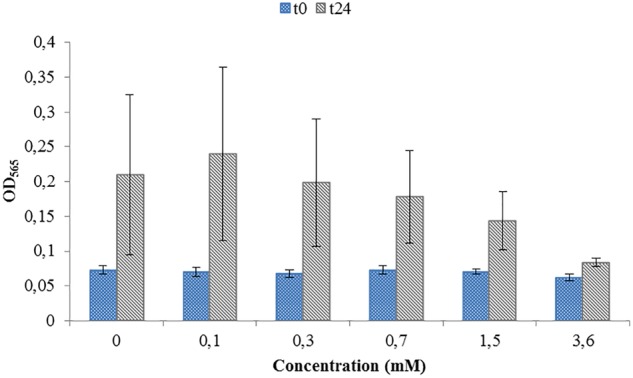
Toxicity test for a range of Potassium ferricyanide used during *M. magneticum* cultivation. Same conditions as in Figure 1. Optical density (OD_565_) measured immediately after the inoculation (t0, blue bars) and after 24 h of cell cultivation (t24, gray bars). Error bars represent the standard deviation of the three repetitions.

Toxicity test were necessary in order to see if the ORP-setting chemicals influence the growth of AMB-1 cells. At this first stage we did not concentrate on the effect of ORP on magnetosome formation, but on the maximal concentration of the chemical that can be used without inhibiting cellular growth. We observed that both titanium (III) citrate and potassium ferricyanide inhibit but do not stop cell growth at high concentration (Figure 1, 2). After the addition of 0.22 mM titanium (III) citrate, we observed a reduction of the growth rate of 17% after 24 h, in comparison to the untreated control. At the concentration of 15 mM of titanium (III) citrate, the growth rate decreased by 51%. In contrast, for the oxidizer, no major effects were measured with the addition of 0.1 mM potassium ferricyanide after 24 h whereas; whereas a 60% decrease was recorded at the concentration of 3.6 mM.

**FIGURE 3:**
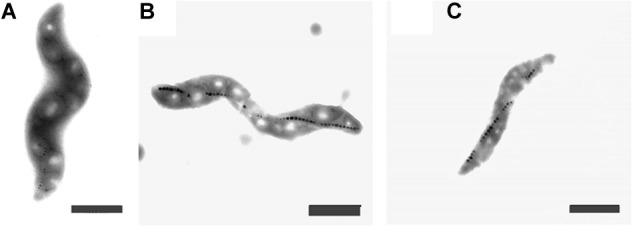
Transmission Electron Images of *M. magneticum* cells after 24 h of cultivation: **(A)** with potassium ferricyanide (3.8 mM); **(B)** without any additions of chemicals mV; **(C)** with Titanium (III) citrate (1.5 mM), Scale bar 1 μm.

The cells grown either with potassium ferricyanide or titanium (III) citrate did not exhibit any change in the external cellular morphology. In both cases, the cells of *M. magneticum* exhibited a short spiral shape, typical for this strain (see Figure 3) and they all actively swim under the microscope. Effects on magnetosome formations were not analyzed during these experiments.

During experiments performed in the bioreactor, titanium (III) citrate and potassium ferricyanide were added continuously via titration systems. The final culture volume was 13 L. During the 42 h cultivation, around 200 mL of either titanium (III) citrate or potassium ferricyanide was necessary to keep ORP either at either -500 or at 0 mV. According to that, the maximum and final concentration set at 0.45 mM of titanium (III) citrate and 0.1 mM of potassium ferricyanide. Comparing these values with our tests, we can therefore assume that the concentration of ORP-setting chemicals will not affect bacterial growth in a large extent.

### Control Experiments Without ORP Adjustment

**FIGURE 4:**
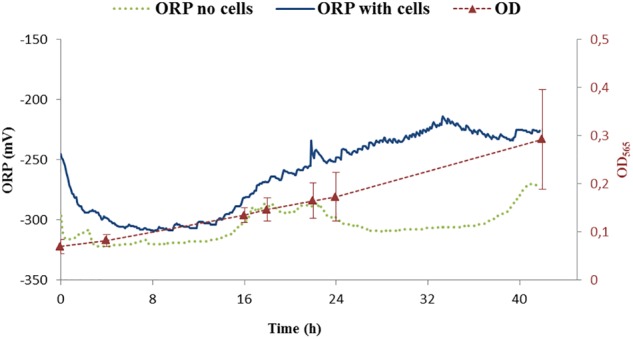
ORP continuous measurements of two controls. One was performed with cells (ORP – dark blue) while the second one was done without cells (ORP – green points). Growth of cells is shown as optical density measurement at the wavelength 565 nm (OD_565_). OD_565_ is shown as red triangles connected with dashed red line as a guide for the eye.

The ORP was controlled by a feedback system of the bioreactor. The ORP change in the growth medium was compared between the medium without cells and with the inoculated medium, the latter being further cultivated for 42 h. The reported ORP values of control experiment in the medium with cells (shown in Figure 4, dark blue line) were first decreasing from –250 to –300 mV in about 1 h, settling for about 15 h before increasing back to –250 mV over time. Below, we will call this condition -250 mV even if the ORP is not controlled by the feedback loop of the bioreactor.

The measurement of the ORP value in the medium without cells settled around -300 mV (Figure 4, green points). Additionally, it was observed that the ORP in the blank medium was probably influenced by the daily light cycle with an increase of around 20 mV for daylight conditions with respect to darkness (i.e., night) indicating potential photochemical reactions taking place in the medium. For our purposes, these changes were considered, when compared to the difference in ORP at which the experiments were performed and thus not further considered: the variation of ORP observed due to light cycle (±20 mV) is around 10 times lower than the experimental controlled ORP variation (±250 mV). Studying the effect of photoactive compounds on the growth of the bacteria and/or studying phototrophic properties of MTB will be of interest in the future.

### Cell Properties

**FIGURE 5:**
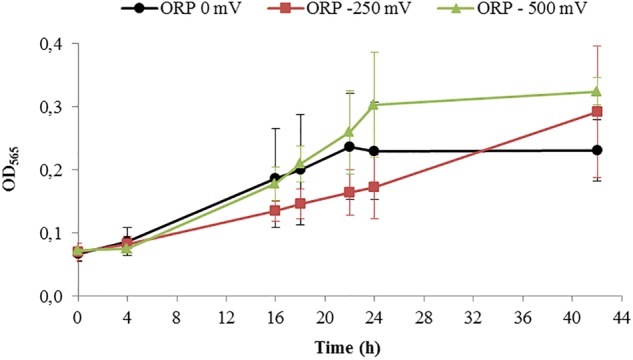
Growth of *Magnetospirillum magneticum* incubated at controlled ORP of 0 mV (black points), -250 mV (red squares) and -500 mV (green triangles), lines as guide for the eyes.

After running the control experiments, we assessed different experiments run at –500 mV, control experiments (around –250 mV as shown above), and 0 mV. Starting with similar cell density, the final OD_565_ was higher for ORP -500 mV in comparison to 0 mV (Figure 5).

Results of the cell length measurements (Table 1) show an abnormal cell elongation for the two tested conditions (0 and 500 mV) compared to regular lengths showed by the control experiments with unclear origin. It is speculated that bacteria become longer in conditions of stress as shown in the literature (Young, 2006).

**Table 1:** Averaged results of the cell growth experiments in the bioreactor at ORP 0, -250, and -500 mV after 42 h of cultivation.

	ORP 0 mV	ORP -250 mV	ORP -500 mV
OD_565_	0.238 (± 0.069)	0.292 (± 0.104)	0.324 (± 0.021)
C_mag_	0.460 (± 0.072)	0.660 (± 0.326)	0.750 (± 0.255)
Cell length (μm)	8.68 (± 2.73)	3.96 (± 2.73)	6.25 (± 5.13)
Iron content (mg^∗^ g^-1^ dry weight)	7.4 (± 1.9)	11.4 (± 2.9)	12.8 (± 3.4)

*Optical density (OD_*565*_), cell magnetism (C_*mag*_), and iron content per dry weight are reported.*

The pellets of cells were collected by centrifugation and the iron content was measured by ICP-OES in order to understand how much iron was accumulated in the cells during the reaction. The results of the iron content in pellets indicated that the iron concentration in the cells (mg g^-1^ dry weight) increased during bacterial growth. The highest intracellular iron accumulation after 42 h was reported at ORP -500 mV, while the lowest at 0 mV, which confirms the hypothesis that AMB-1 strain forms magnetosomes preferably under reduced conditions (Yang et al., 2001). The differences obtained for optical density, cell magnetism and iron content per dry weight under reduced and oxidized conditions are summarized in Table 1.

### Magnetosome Properties

**FIGURE 6:**
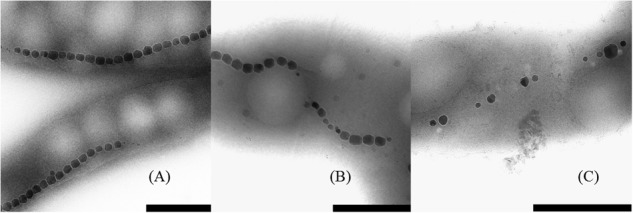
Close up images of the magnetosome chains. **(A)** ORP -500 mV, **(B)** ORP -250 mV, and **(C)** ORP 0 mV (scale bar 500 nm).

With TEM imaging, it was possible to count the number of magnetosomes per cell and to measure the size of the magnetosomes. The results are expressed as number of magnetosome per μm of cell to avoid bias due to differences in the cells length as reported above. For particle size measurements, the major and minor axes of the crystals were analyzed and the surface of the ellipse was calculated. A clear trend in magnetosomes amount and size is visible among tested conditions; at ORP -500 mV the highest magnetosome size and number was observed, at ORP -250 mV magnetosomes exhibited similar values, while at ORP 0 mV the lowest size of particles and lowest amount of magnetosomes was observed. The results are summarized in Table 2. In Figure 6, representative TEM images of magnetosomes chains are shown.

**Table 2:** Mean magnetosome size measurements analyzed by X-ray diffraction, compared with magnetosome diameter obtained via transmission electron images analysis, measured at ORP 0, -250, and -500 mV.

Growth conditions	Nanoparticles diameter (x-ray diffraction) (nm)	Magnetosome diameter (image analysis) (nm)	Number of magnetosomes μm cell length^-1^
ORP 0 mV	31.5 (±1.3)	34.5 (±12.3)	9.1 (±1.9)
ORP -250 mV	37.2 (±0.8)	46.7 (±15.7)	8.6 (±1.5)
ORP -500 mV	37.2 (±0.6)	50.8 (±13.6)	5.48 (±1.3)

*Measurements of the number of magnetosomes, obtained via analysis of transmission electron microscope images.*

Briefly, the magnetosome sizes we obtained at ORP -500 and -250 mV are 50.8 ± 13.6 and 46.7 ± 15.7 nm respectively, in agreement with a previous study (Li and Pan, 2012). However, it is important to mention that many small particles might have been excluded from the measurements due to the filtering the particle area at 225 nm^2^ particle area and at a roundness of 0.7. This issue had a strong influence, especially at ORP 0 mV, where the average size of magnetosomes was 34.5 ± 12.3 nm and where in particular many magnetosomes were underdeveloped and certainly have not reached mature size.

The mean magnetosome size was also analyzed with XRD. The trend in the decrease of particles size obtained by both methods is similar for ORP 0 mV. The diameter of nanoparticles measured via X-ray is 31.5 ± 1.3 nm while size of nanoparticles measured from TEM images is 34.5 ± 12.3 nm. The size of nanoparticles synthesized at -250 mV (46.7 ± 15.7 nm) and at ORP -500 mV (50.8 ± 13.6 nm) showed higher values than the sizes obtained via XRD; ORP -500 mV: 37.2 ± 0.6 nm; ORP -250 mV: 37.2 ± 0.8 nm. This confirmed the hypothesis that ORP 0 mV caused a decrease in the size of magnetosomes. The comparison between these two techniques is shown in Table 2. We performed an analysis of variance (ANOVA) on the magnetosome sizes. The tests yielded a significant variation among conditions [*F*(2; 1197) = 72.84, *p* < 2e-16]. A *post hoc* Tukey test showed that all the groups differ significantly at *p* < 0.001.

## Discussion

In this work, the formation of magnetosomes was monitored during cultivation in a bioreactor at different ORP values. ORP was varied between -500 and 0 mV. Previous studies only analyzed the effect of oxygen partial pressure as an oxidizer, where variations of ORP values were the consequence of different oxygen concentrations (Li and Pan, 2012; Moisescu et al., 2014), making this study the first one to disentangle ORP and oxygen concentration. Since the experiments were performed under strictly controlled anaerobic conditions, it was possible to exclude oxygen coming from the air and use other chemical agents to modify the redox state of the medium. The redox control during the cultivation of AMB-1 cells under reduced and oxidized conditions was successfully achieved by using either titanium (III) citrate or potassium ferricyanide.

### Effect of Redox-Active Chemicals on Cells Morphology and Growth

In order to disentangle the effect of ORP from the effect of redox-active chemicals (titanium (III) citrate and potassium ferricyanide), we performed preliminary experiments and toxicity tests, which showed that the concentration of these chemicals, at which we later use them, is not dramatically affecting the cells. Even at the highest, tested concentrations, 15 and 3.6 mM for titanium (III) citrate and potassium ferricyanide respectively, cells remained motile. They exhibited slower growth but it was still observable. Other studies done by Li and Pen showed that under anaerobic conditions and after 40 h growth, the cell amount calculated as cells OD was much lower (OD_600_ ca. 0.05), when compared to cells grown in our bioreactor for 42 h (Li and Pan, 2012). Even aerobic conditions, which usually induce cell growth, reached lower OD values (OD_600_ < 0.2) than cell grown in our system at all used conditions; at 0 mV, OD_565_ = 0.238; at -250 mV, OD_565_ = 0.292, at -500 mV, OD_565_ = 0.324). More research will thus be necessary to clarify the origin of the recorded cell elongation.

### Effect of ORP on Iron Accumulation

Iron accumulation measurements showed an inhibition of the biomineralization process at ORP 0 mV, while it was enhanced at -500 mV. Possible reasons for such behavior could be connected to the chemical behavior of iron in aquatic solutions. Magnetite synthesis requires a 2:1 Fe(III):Fe(II) ratio. At lower levels of ORP (the -250 and -500 mV conditions), the synthesis of magnetite (Fe_3_O_4_) at pH around neutrality is thermodynamically favored. At higher ORP levels, other iron oxide phases like Fe_2_O_3_ are advantaged (Bell et al., 1987). The values of iron uptake appeared to be in the same range as previous studies on *M. magneticum* AMB-1, when compared on the laboratory scale. Heyen and Schüler showed that in large 4 L scale fermenter the iron content, after AMB -1 growth, reached 9.8 mg × g^-1^ dry mass. Our experiment at four time bigger scale (16L) showed that the amount of iron was much higher, reaching 12.8 mg × g^-1^ dry mass. This increase showed that AMB-1 strain is even better candidate for the production of magnetosomes than already, widely used MSR – 1, on the technical scale, under reduced fermentation conditions.

### Effect of a Chemically Induced ORP vs. Effect of Oxygen

Our experiment also confirmed the results of similar previous works, where biomineralization was studied in relationship to oxygen concentration. Schüler and Baeuerlein (1998) observed for example magnetite formation occurring only in a narrow range of low oxygen concentrations for *Magnetospirillum gryphiswaldense*. Popa et al. (2009) conducted a similar experiments with *M. magneticum* strain AMB-1 and also reported biomineralization inhibited at high oxygen concentrations, while restored with the addition of a reducing agent. In Table 3, a comparison between data obtained in this study with results obtained by Heyen and Schüler (2003) is shown. It exemplified that at lower oxygen concentration (0.25 mbar partial pressure); an iron accumulation is observed, which origin remains unclear. Once the oxygen partial pressure increased, iron accumulation was reduced with values similar to those in our experiment. Interesting would be to know which ORP values correspond to the values expressed by Heyen and Schüler (2003), in order to have fully comparable results. A logical extension of the here presented study would be the investigation of the mechanism behind the observed phenomenon. Although *M. magneticum* has been shown to possibly take up both Fe (II) and Fe (III) (Faivre et al., 2007), the first is assume to be assimilated by passive absorption, while Fe (III) are actively taken up (Schüler and Baeuerlein, 1996; Amor et al., 2018). As a result, we can speculate that the effect of ORP could be to modify the ratio between the two oxidation states, thereby influencing the action of the MTB redox proteins, resulting in a slower and/or more energetically-costly biomineralization process.

**Table 3:** Iron content measurements reported for ORP conditions, normalized according to dry weight (left column).

ORP experiments		Heyen and Schüler, 2003	
ORP	Iron content (mg^∗^g^-1^ dry weight)	Oxygen partial pressure (mbar)	Iron content (mg^∗^g^-1^g dry weight)
–500 mV	12.8 (±3.4)	0.25	9.8
–250 mV	11.4 (±2.9)	2	7.9
0 mV	7.4 (±1.9)	10	5.7

*Iron content measurements reported by Heyen and Schüler (2003) for AMB-1 strain (right column) reported for different oxygen partial pressure.*

### Effect of ORP on Size and Amount of Magnetosomes

Li and Pan (2012) showed that the average magnetosome size in AMB-1 cells grown under anaerobic conditions was 41.5 ± 15.0 nm with the average shape factor, determined from TEM images, of 0.78. Comparing our results of image analysis of magnetosomes, those done at 0 and -250 mV appear to be in line with those measured by Li and Pan. But magnetosomes obtained at the ORP – 500 mV are 10 nm bigger. The amount of magnetosomes obtained per cell is also higher when compared to studies of Yang et al. (2013) or Moisescu et al. (2011). The average amount of particles per cell reached 28 and 39 magnetosomes per cell, respectively. In our case, the amount of particles was calculated per μm due to enourmous cells elongation and it reached 6 magnetosomes. When calculated per cell, we reached a number of 94 magnetosomes per cell at –500 mV. In TEM size analysis, only a few particles were measured, whereas in the x-ray data analysis bulk dimension was determined, which gives a better overview about the actual size distribution in the whole sample. TEM observations are used as a double proof of the size and can additionally give raise of the magnetosome shape and the structure.

### Other Parameters Related to ORP and Their Potential Effects

Beside the cell growth, magnetosome formation and iron dynamics, possible effects could potentially arise from the chemicals used too. Moreover, citric acid in bacteria has been reported to act as an exogenous siderophore (Silva et al., 2009) and *M. magneticum* have been reported to possibly secrete siderophore 3,4-dihydroxybenzoic acid for Fe (III) complexation (Calugay et al., 2004). The ability to bind ferric iron leads to the formation of citrate correlated siderophores such as rhizoferrin and staphyloferrin A, where the structure of the chelating unit preserves the citric acid structure (Johnstone and Nolan, 2015). This molecular mechanism would cause higher iron accumulation on the surface of the cells and may also be partly responsible for the enhancement of magnetosome formation at -500 mV. With the addition of potassium ferricyanide, the magnetosomes formation was disrupted at 0 mV. A previous study of Bagramyan et al. (2000) showed how a change in ORP in the growth medium of bacteria, specifically *E. coli*, influences the membrane transport mechanisms, in particular H^+^ and K^+^ fluxes trough the membrane. We hypothesize, that the oxidative stress might also reduce the iron flux across the membranes. It should be also taken into a consideration that the ORP of the medium is the outcome of a chemical mixture. That is why it is important to remember that there might me other reducing species affecting the ORP and in consequence influencing the ions flux. For example organic species, such as phenols or other metal ions could influence intracellular states, especially as environmental pollutants (Enache and Oliveira-Brett, 2011; Liu et al., 2013). It was shown that AMB–1 can mineralize tellurium nanorods separately from its magnetite crystals as a new method for recovering this rare element from the environment (Tanaka et al., 2010). Other organisms use biomineralization to detoxify pollutants, such as Rhodanobacter A2-61 that forms intracellular uranium – phosphate complexes (Sousa et al., 2013).

## Summary and Conclusion

In summary, the results here highlight the differences between the bacteria grown at different ORP. These results are useful for the elucidation of the relationship between physico-chemical conditions of the growing medium and the biomineralization processes within the bacteria, showing that it is a variation in the external ORP, not specifically of oxygen, which ultimately is responsible for the observed effect on biomineralization. Finally, we show that ORP monitoring and tuning is of interest for the production of magnetosome in high yields. This is a prerequisite for the application of these biological particles with high added value in nano- and biotechnologies such as cancer treatment and hyperthermia (Alphandéry et al., 2011), for MPI research (Kraupner et al., 2017) or as contrast agent in magnetic resonance imaging (Taukulis et al., 2015). However, all these applications require several mg of magnetosmes. With current yields obtained from bioreactors (about 1 mg per L of culture) and the necessary amount requested for the above cited application (tens of mg of magnetosome per patient), there is an urgent need for the development of biotechnological processes leading toward the high yield production of magnetosomes. We show here that a high amount of particle is obtained at low ORP. Therefore; coupling current processes where high cellular yield can be obtained first, with our approach showing that low ORP are favorable for high yield production of magnetosomes, we expect that the development of magnetosome based applications will be simplified.

## Author Contributions

AO-W and DF conceived the project. GS, AO-W, and VR conducted the experiments. AO-W and GS contributed equally to this work. DF supervised the work. All authors discussed the results of the experiments and contributed to the final manuscript that was initially drafted by AO-W and GS. All authors approved the submitted version.

## Conflict of Interest Statement

The authors declare that the research was conducted in the absence of any commercial or financial relationships that could be construed as a potential conflict of interest. The handling editor declared a past co-authorship with one of the authors DF.

## Abbreviations

C_mag_: the coefficient of magnetically induced differential light scattering
FSM: Flask Standard Medium
MTB: Magnetotactic Bacteria
OATZ: Oxic-Anoxic Transition Zone
OD: Optical Density
OD_565_: Optical Density measured at 565 nm
OD_max_: Optical Density measured with magnet placed perpendicular to the light beam
OD_min_: Optical Density measured with magnet placed parallel to the light beam
ORP: Oxidation – Reduction Potential
