# Supplementary material for: Reducing Conditions Favor Magnetosome Production in Magnetospirillum magneticum AMB-1
Source: Front Microbiol. 2019 Mar 29;10:582. doi: 10.3389/fmicb.2019.00582 (PMC6450187; doi:10.3389/fmicb.2019.00582)
Supplement: Supplementary file 1 [file Data_Sheet_1.docx]

Supplementary Material

**Reducing conditions favor magnetosome production in *Magnetospirillum magneticum* AMB-1**

Agata Olszewska-Widdrat^1,2^, Gabriele Schiro^1,3^, Victoria E. Reichel^1,4^,Damien Faivre^1,5*^

*** Correspondence:** Corresponding Author: damien.faivre@cea.fr


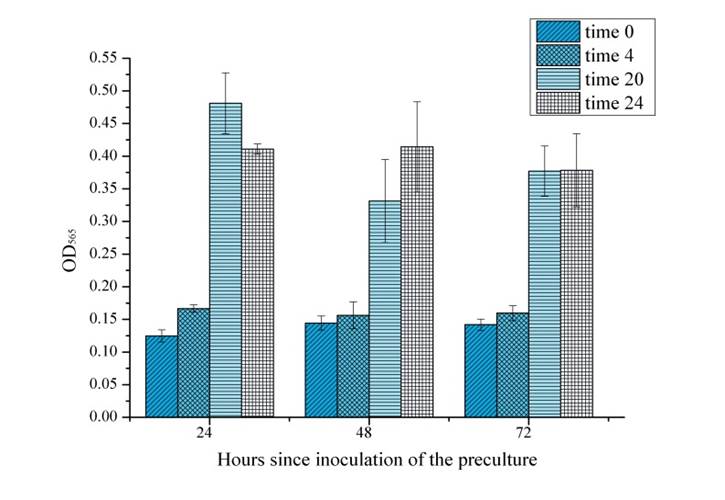


**Supplementary Figure 1.** A preculture was prepared in 500 mL flask filled with 250 mL of iron depleted FSM medium, sealed under aerobic conditions. Bacteria were inoculated by injection through the stopper. The preculture was incubated for 24 hours, under shaking conditions (100 rpm). After that time 10 mL of the preculture was injected into the new flask and further cultivated. The initial preculture was continuously cultivated and a second inoculum was prepared after 48 hours and a third one and final one, after 72 hours. OD_565_ of new subcultures was measured immediately after the inoculation, and after 4, 20 and 24 hours respectively.


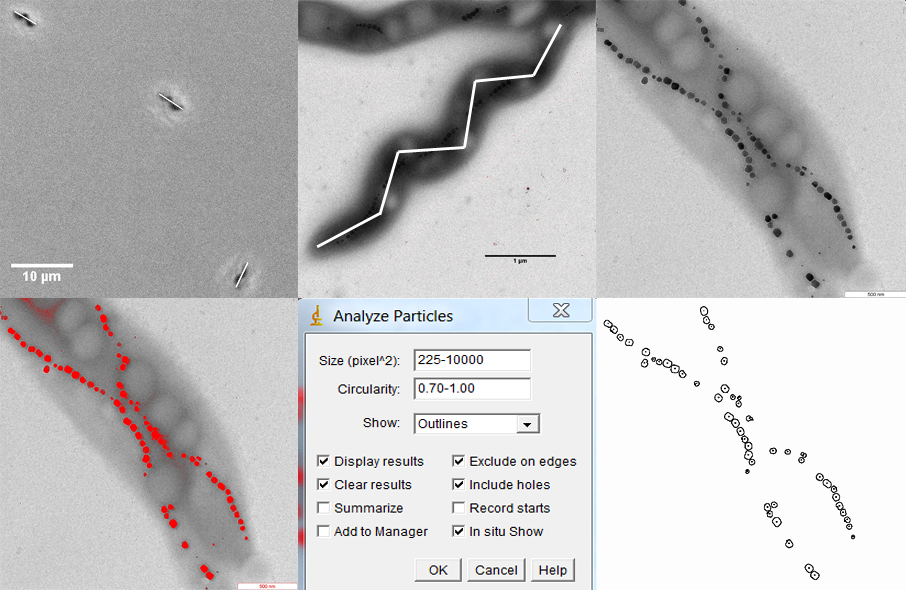


(a)

(b)

(c)

(e)

(d)

(f)

Supplementary Figure 2. Size measurements. Panel “a” represents the method utilized for length measurements with the optical microscope. The length of the line drawn between the two poles of a bacterium was measured. Panel “b” (scale bar 1 µm) shows how the length of a bacterium was measured in TEM images. The length of the white line drawn in the middle of the bacterium was taken as the measurement. In panels “c, d, e, f” measurements of particles size using the software “imageJ” are shown: panel “c” represents one of the picture analyzed, in panel “d” the threshold was set, in panel “e” the settings for the measurements were inserted, while panel “f” shows the outlines of the particles selected for measurements (panel c, d, f, scale bar = 500 nm).

Supplementary Figure 3. Constant pH measurements of medium in the bioreactor (green line) without cells. Constant measurements of pH of the medium containing preculture, that was subsequently grown over 42 hours (blue line). Growth of cells is shown as optical density measurement at the wavelength 565 nm (OD_565_). OD_565_ is shown as red triangles connected with dashed red line.
